# Denoising single-cell ATAC-seq data with Shannon entropy

**DOI:** 10.64898/2026.10.20.760589

**Published:** 2026-10-23

**Authors:** Shiyi Yang, Kevin An, Juliana J. Lee, Jase Gehring, Sina Booeshaghi, Haiyan Huang, Aaron Streets

## Abstract

Single-cell Assay for Transposase Accessible Chromatin with sequencing (scATAC-seq) is widely used to investigate chromatin accessibility but experiments often contain empty droplets, droplets containing damaged nuclei or cellular-debris. Accurately filtering high-quality nuclei is important as noisy droplets contribute reads that can distort downstream analysis and obscure rare cell population. Current approaches often rely on read depth and or predefined genomic features that are not exactly the same as chromatin accessibility landscape. Here, we present a Shannon entropy-based method that filters high-quality nuclei more robustly by quantifying genome-wide accessibility complexity. This approach improves signal-to-noise ratio, increases sensitivity for accessible-region detection, and enables more reliable downstream inference in scATAC-seq studies.

